# Towards prehospital risk stratification using deep learning for ECG interpretation in suspected acute coronary syndrome

**DOI:** 10.1136/bmjhci-2024-101292

**Published:** 2025-06-06

**Authors:** Jesse P A Demandt, Thomas P Mast, Konrad A J van Beek, Arjan Koks, Marieke C V Bastiaansen, Pim A L Tonino, Marcel van ’t Veer, Frederik M Zimmermann, Pieter-Jan Vlaar

**Affiliations:** 1 Department of Cardiology, Catharina Hospital, Eindhoven, Netherlands; 2 GGD Brabant-Zuidoost, Eindhoven, Noord-Brabant, Netherlands; 3 Department of Biomedical Engineering, Eindhoven University of Technology, Eindhoven, Netherlands; 4 Department of Cardiology, Sint Antonius, Nieuwegein, Utrecht, Netherlands

**Keywords:** Deep Learning, Artificial intelligence

## Abstract

**ABSTRACT:**

**Objectives:**

Most patients presenting with chest pain in the emergency medical services (EMS) setting are suspected of non-ST-elevation acute coronary syndrome (NSTE-ACS). Distinguishing true NSTE-ACS from non-cardiac chest pain based solely on the ECG is challenging. The aim of this study is to develop and validate a convolutional neural network (CNN)-based model for risk stratification of suspected NSTE-ACS patients and to compare its performance with currently available prehospital diagnostic tools.

**Methods:**

For this study, an internal training cohort and an external validation cohort were used, both consisting of suspected NSTE-ACS patients. A CNN (ECG interpretation by CNN (ECG-AI)) was trained and validated to detect NSTE-ACS. The diagnostic value of ECG-AI in detecting NSTE-ACS was compared with on-site ECG analyses by an EMS paramedic (ECG-EMS), point-of-care troponin assessment and a validated prehospital clinical risk score (prehospital History, ECG, Age, Risk factors and POC-troponin (preHEART)).

**Results:**

A total of 5645 patients suspected of NSTE-ACS were included. In the external validation cohort (n=754), 27% were diagnosed with NSTE-ACS. ECG-AI had a better diagnostic performance than ECG-EMS (area under the curve (AUROC) 0.70 (0.66 to 0.74) vs AUROC 0.65 (0.61 to 0.70), p=0.045) for diagnosing NSTE-ACS. The overall diagnostic accuracy of preHEART was AUROC 0.78 (0.74 to 0.82) and superior compared with ECG-AI (p=0.001). Incorporating ECG-AI into preHEART led to a significant improvement in diagnostic performance (AUROC 0.83 (0.79 to 0.86), p<0.001).

**Discussion:**

Correctly identifying patients who are at low risk for having NSTE-ACS is crucial for optimal triage in the prehospital setting. Recent studies have shown that these low-risk patients could potentially be left at home or transferred to a general practitioner, leading to less emergency department overcrowding and lower healthcare costs. Other studies demonstrated better overall diagnostic performance compared with our artificial intelligence (AI) model. However, these studies were aimed at a study population with a high prevalence of occlusive myocardial infarction, which could explain the differing levels of diagnostic performance.

**Conclusion:**

Integrating AI in prehospital ECG interpretation improves the identification of patients at low risk for having NSTE-ACS. Nonetheless, clinical risk scores currently yield the best diagnostic performance and their accuracy could be further enhanced through AI. Our results pave the way for new studies focused on exploring the role of AI in prehospital risk-stratification efforts.

**WHAT IS ALREADY KNOWN ON THIS TOPIC**

- Emergency medical services studies have shown that prehospital risk stratification in suspected non-ST-elevation acute coronary syndrome (NSTE-ACS) patients can be improved using clinical risk scores with point-of-care troponin. However, the potential of artificial intelligence (AI) to further improve the diagnostic performance of these clinical risk scores has not yet been explored.

**WHAT THIS STUDY ADDS**

- This study demonstrates that the integration of AI into clinical risk scores significantly enhances their predictive accuracy. Notably, sensitivity and negative predictive value for identifying low-risk NSTE-ACS patients can be improved through AI incorporation.

**HOW THIS STUDY MIGHT AFFECT RESEARCH, PRACTICE OR POLICY**

- With many countries either considering or already implementing clinical risk scores for suspected NSTE-ACS in the prehospital setting, our findings suggest that the incorporation of AI can enhance the safety and effectiveness of these tools. This study lays the groundwork for future research aimed at evaluating the role of AI in prehospital risk stratification.

## Introduction

 Chest pain is one of the most common reasons for consulting emergency medical services (EMS) and an ECG should be obtained at first medical contact.^1 2^ Based on the initial prehospital ECG, patients can be differentiated into two working diagnoses: chest pain with ST segment elevation (STEMI) or chest pain without persistent ST segment elevation (suspected non-ST-elevation acute coronary syndrome (NSTE-ACS)).^1^,^4^ In case of suspected NSTE-ACS, it is challenging in the prehospital setting to distinguish patients who have true NSTE-ACS from those who are experiencing non-cardiac chest pain, and this hampers further prehospital triage.^5^

Prehospital ECG interpretation by EMS paramedics is complicated by several challenges. First of all, the ECG may be normal in more than one-third of NSTE-ACS patients. In addition, an important part of the NSTE-ACS patients has only subtle or transient ischaemic ECG alterations and the presence of a bundle branch block, a paced rhythm or previous myocardial injury precludes the interpretation of ischaemic ECG alterations.^1^ Finally, inter-observer variability is common in ECG interpretation and is dependent on the level of experience.^6 7^ These aforementioned factors result in a modest performance of the ECG, as a diagnostic tool alone for suspected NSTE-ACS in the prehospital setting.^5 8^ Nevertheless, the importance of the ECG is underscored by its incorporation in current (pre)hospital risk scores and triage algorithms, such as the Global Registry of Acute Coronary Events for in-hospital mortality, History, Electrocardiogram, Age, Risk, Troponin (HEART) and Thrombolysis in Myocardial Infarction (TIMI) risk scores.^59^,^15^

Due to a lack of adequate diagnostic tools in the prehospital setting, all suspected NSTE-ACS patients are currently transferred to the nearest hospital, with or without percutaneous coronary intervention facilities, for further diagnostic work-up.^8 12^ Of these suspected NSTE-ACS patients, the majority does not have underlying life-threatening pathology and can be discharged the same day.^10 11^ Recent studies have shown that performing adequate prehospital risk stratification and triage decisions with clinical risk scores can lead to less emergency department (ED) overcrowding by leaving patients at low risk for having NSTE-ACS at home or transferring them to the general practitioner, decreasing time to revascularisation and hospital discharge in patients with diagnosed NSTE-ACS, and lowering healthcare costs.^10^,^1216^

The recent advances in artificial intelligence (AI) models, specifically convolutional neural networks (CNNs), in the field of ECG interpretation have demonstrated highly encouraging results. These models are capable of detecting subtle ECG patterns, potentially enhancing the accuracy of ECG interpretation and eliminating inter-observer variability.^17^,^21^ Consequently, such models can potentially play a significant role in prehospital risk stratification of NSTE-ACS patients with occluded coronary arteries.^22 23^ The aim of this study is to develop and validate a neural network to identify patterns within the ECG indicative of NSTE-ACS. In addition, to compare the diagnostic performance of this model to the diagnostic performance of prehospital ECG interpretation by EMS paramedics and other established diagnostic tools in the prehospital setting (point-of-care (POC) assessment of the biomarker troponin and a validated clinical risk score).

## Methods

### Study setting and design

For this study, an internal training cohort and an external validation cohort were used. Data for the internal training cohort were extracted from the Catharina Hospital (Eindhoven, the Netherlands), a tertiary care centre with 24/7 cardiac surgery and primary PCI facilities. Individuals eligible for inclusion in this study were patients who presented at the ED between 1 June 2015 and 31 December 2020 and, on evaluation, received a diagnosis of either ‘NSTE-ACS’ or were diagnosed with ‘non-cardiac chest pain’ based on the ICD-10 registration code. Raw data for the standard 12-lead 10 s ECG and the final diagnosis were collected from these patients, with the first recorded ECG on admission in the ED selected for analysis. ECGs were acquired at a sampling rate of 5000 Hz using a GE-Marquette ECG machine (Marquette, Wisconsin, USA).

Data from the TRIAGE-ACS study were used for the external validation cohort. The design and results of the study have been published previously.^12 24^ In summary, the TRIAGE-ACS study is a prehospital, multicentre, prospective cohort study conducted in the Netherlands, where patients suspected of NSTE-ACS were enrolled by EMS paramedics and a standard ECG was obtained according to EMS protocols. Besides on-site ECG analyses, also POC-troponin assessment and a validated clinical risk score (the prehospital History, ECG, Age, Risk factors and POC-troponin (preHEART) score) were calculated to guide prehospital risk assessment and triage.^5 12^ Patients were excluded if there was STEMI on the prehospital ECG or fulfilled one of the very high-risk criteria according to the European Society of Cardiology (ESC) and American Heart Association/American College of Cardiology (AHA/ACC) guidelines (eg, haemodynamic unstable, cardiogenic shock, ongoing chest pain refractory to medical treatment).^1 2^ The complete list of inclusion and exclusion criteria is provided in online supplemental table S1.

All prehospital ECGs from the TRIAGE-ACS study were automatically stored in CODESTAT data review software (V.12.0, Stryker Corporation, Kalamazoo, Michigan, USA). The first ECG captured by EMS paramedics either at the scene or during transportation was extracted. For the purpose of this study, we used the raw data of the standard 12-lead 10 s ECG data, also stored at 5000 Hz, for analysis together with the diagnosis after evaluation on the ED. Adjudication of the diagnosis of NSTE-ACS is performed by applying current ESC guidelines and the fourth universal definition of myocardial infarction.^25 26^ NSTE-ACS is defined as non-ST-elevation myocardial infarction (NSTEMI) or unstable angina pectoris. The final diagnosis was checked by two independent and blinded medical doctors.

### Preprocessing of the ECG

The data from the 12-lead, 10 s ECG were converted into median beats using both the Muse and CODESTAT software. These software packages automatically provided us with median beats per lead. The median beat data were constructed by aligning all QRS complexes of the same shape (eg, excluding premature ventricular complexes) and generating a representative QRS complex by taking the median voltage. The generation of the median beat reduces noise by averaging all beats over the 10 s ECG and aligning each complex to minimise variations, thereby improving measurement accuracy. These data were then used as input for the AI model.

### Model development

Given the disparities between the internal training cohort and external validation cohort in terms of timing and setting, we adopted a sevenfold cross-validation approach (online supplemental figure S1). Our model, a variant of a CNN named DeepArrnet, was used to diagnose NSTE-ACS within the ECG. DeepArrnet is designed to use both temporal and pointwise convolution techniques to extract features from ECG data for classification purposes.^27^

The training and testing datasets were formed by combining the entire internal training cohort and 6/7ths of the external validation cohort. From this combined dataset, a random sample of 80% was allocated for model training, and the remaining 20% was used for testing. To prevent overfitting, the number of training epochs was controlled by an early stopping mechanism. The final model underwent external validation using a hold-out set, comprising the unused 1/7th of the external validation cohort, to assess performance metrics. This process was iterated seven times, with each iteration training, testing and validating a new model, ensuring that every ECG from the external validation cohort was included exactly once in a hold-out set. In the final layer, we applied a softmax function to transform the model’s output into a numerical range between 0 and 1 (ECG-AI). The model was constructed using Python’s Keras framework (V.3.7.3) (online supplemental figure S2).

### Model comparison

The diagnostic performance of the CNN-based interpretation by the AI model (ECG interpretation by CNN (ECG-AI)) was compared with prehospital ECG interpretation by EMS paramedics (ECG-EMS), POC assessment of the biomarker troponin-I and the preHEART score. For interpreting the ECG, as part of scoring the preHEART score, the EMS paramedics were trained to score the ECG in three risk categories: low risk (normal ECG), intermediate risk (abnormal ECG with repolarisation abnormalities; including left bundle branch block, right bundle branch block) or high risk for having NSTE-ACS (ECG with specific ischaemic abnormalities; new ST-depression or T-wave inversion). New ST-segment depression was defined as horizontal or down-sloping ≥0.05 mV in two consecutive leads. T-wave inversion as >0.01 mV in two consecutive leads. To gain more insight into the potential differences between the ECG interpretation by EMS paramedics and AI, we additionally transformed the continuous ECG-AI parameter, which ranges from 0 to 1 predicted risk, was transformed into three risk categories: low, intermediate and high. These categories were aligned with the risk stratification used by EMS paramedics in their ECG interpretations. To achieve this, two cut-off points (X and Y) were required. An ECG-AI predicted risk of 0 to X indicated low risk, X to Y indicated intermediate risk, and Y to 1.0 indicated high risk, where X<Y. The transformation of the continuous ECG-AI parameter into three risk categories was performed by testing 4863 combinations of cut-off points. The cut-off combination that yielded the best corresponding area under the curve (AUROC) was selected, acknowledging that the AUROC for a categorised variable is inherently lower than that of the continuous variable. This process was performed solely for comparison purposes.

An ECG-AI predicted risk of 0%–4% was considered low, 4%–29% intermediate and 29%–100% high. This parameter, now consisting of three risk categories, was compared with the three risk categories (low, intermediate and high) assigned by EMS personnel to the ECGs.

For measuring troponin-I, blood was collected on-site via intravenous access by an EMS paramedic and measured using a POC-analyser ‘I-stat’ (Abbott industries, Minneapolis, Minnesota, USA). Analysing the blood sample takes approximately 10 min. The reportable range of the POC-analyser is 0.00–50.00 ng/mL, and 17 µL of blood is required to fill the cardiac troponin-I cartridge. The assay has a limit of detection of 0.02 µg/L and a 99th percentile upper reference limit of 0.08 µg/L.^28^

As a clinical risk score, the preHEART score, derived from the well-known HEART score and optimised and validated for prehospital use, was used.^5 12 29^ The preHEART score can be calculated by EMS paramedics based on five different elements: history, ECG, age, gender and POC troponin-I. The total score ranges between a minimum of 0 points and a maximum of 10 points and depending on this score, the patient can be labelled as low-risk, intermediate-risk or high-risk for having NSTE-ACS (online supplemental table S2). To investigate the effect of AI on a clinical risk score, the ECG-EMS was replaced and ECG-AI was implemented, as a continuous variable, in the preHEART score using logistical regression analysis, resulting in the prehospital History, ECG-AI, Age, Risk factors and POC-troponin (preHEART-AI) score.

### Statistical analysis

Continuous variables are expressed as means±SD or medians (IQRs). Categorical variables are reported as frequencies and percentages. Differences in continuous variables between groups are assessed through the unpaired t-test or Mann-Whitney U test, while differences in categorical data are assessed with the χ^2^ test or Fisher’s exact test. The diagnostic performance is expressed by ROC analysis together with the corresponding AUROC (overall diagnostic performance), sensitivity, specificity, positive predictive value (PPV) and negative predictive value (NPV). The AUROC is compared according to the DeLong method.^30^ Two by two tables are used to calculate the sensitivity and NPV for identification of the lowest risk group for ECG-EMS and ECG-AI, and specificity and PPV for the identification of the highest risk group. Sensitivity and specificity are compared using McNemar’s test. Statistical significance is defined as a two-sided p value <0.05. Analyses were performed by using SPSS V.29.0 and R V.4.3.1 (R Foundation for Statistical Computing, Vienna, Austria).

### Regulation statement

The study is reviewed and approved by the regional Medical Ethics Committee (MEC-U W21.045).

## Results

A total of 5645 patients suspected of NSTE-ACS were included. The internal training cohort consisted of 4891 12-lead ECGs of suspected NSTE-ACS patients, mean age was 63±15 years, 44% were female and 25% (n=1239) were diagnosed with NSTE-ACS after examination in the ED (online supplemental table S3). The external validation cohort consisted of 754 12-lead ECGs of suspected NSTE-ACS patients, mean age was 67±13 years, 45% were female and 27% (n=202) were diagnosed with NSTE-ACS. Additional baseline characteristics are presented in table 1.

**Table 1:** Baseline characteristics

	External validation cohort (n=754)
Age (years), mean±SD	67±13
Female sex, n (%)	340/754 (45)
Time from symptom onset until first ECG by EMS paramedic (min), median (IQR)	130 (60–335)
Duration of symptoms (min), median (IQR)	90 (30–161)
Hypertension, n (%)	427/744 (57)
Family history of ACS, n (%)	257/520 (49)
BMI≥30 kg/m^2^, n (%)	191/620 (31)
Previous ACS, n (%)	224/754 (28)
Systolic blood pressure (mm Hg), mean±SD	148±26
Heart rate (bpm), median (IQR)	73±14
Hb (mmol/L), median (IQR)	8.6 (8.0–9.1)
Creatinine (µmol/L), median (IQR)	78 (68–93)
NSTE-ACS, n (%)	213/754 (28)
GRACE score, mean±SD	109±28

ACS, acute coronary syndrome; BMI, body mass index; BPM, beats per minute; EMS, emergency medical services; GRACE, Global Registry of Acute Coronary Events for in-hospital mortality; Hb, haemoglobin; NSTE-ACS, non-ST-elevation ACS; POC, point-of-care; STEMI, ST-segment elevation myocardial infarction.

**Figure 1:**
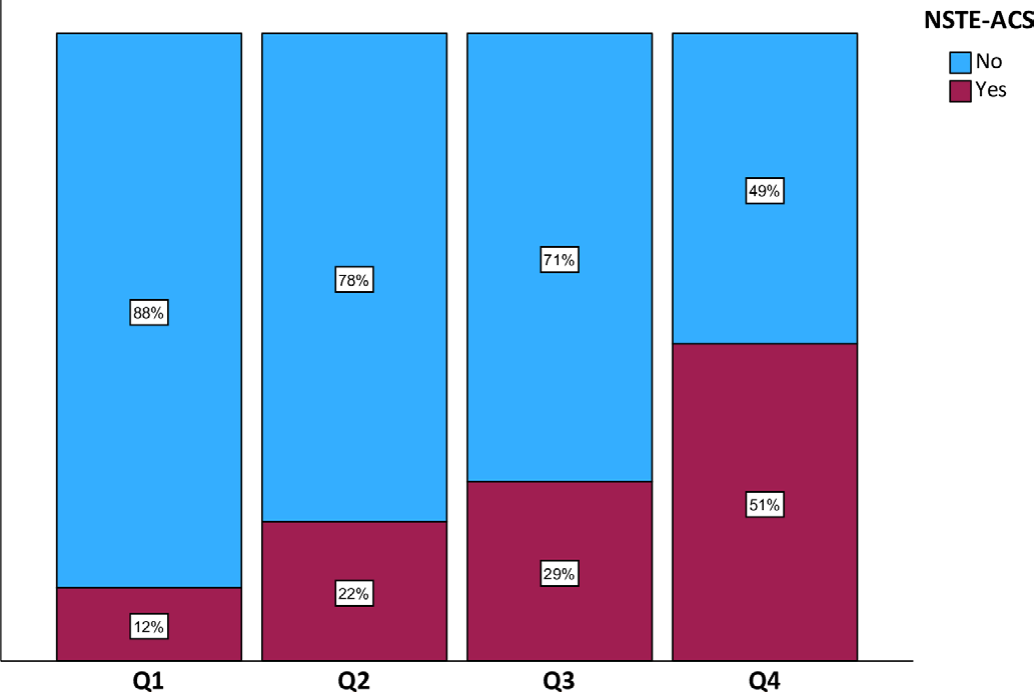
Percentage of NSTE-ACS per quartile for ECG-AI. ECG-AI, ECG interpretation by AI model; NSTE-ACS, non-ST-elevation acute coronary syndrome.

After developing the CNN using the internal training cohort, ECG-AI was validated in the external validation cohort. ECG-AI was computed as a continuous variable between 0 and 1. Figure 1 shows the per-quartile performance of the ECG-AI score in the validation cohort. Patients with ECG-AI scores in the highest quartile have a 51% chance of having NSTE-ACS. In the lowest quartile, exclusion of ACS was found in 88%.

**Figure 2:**
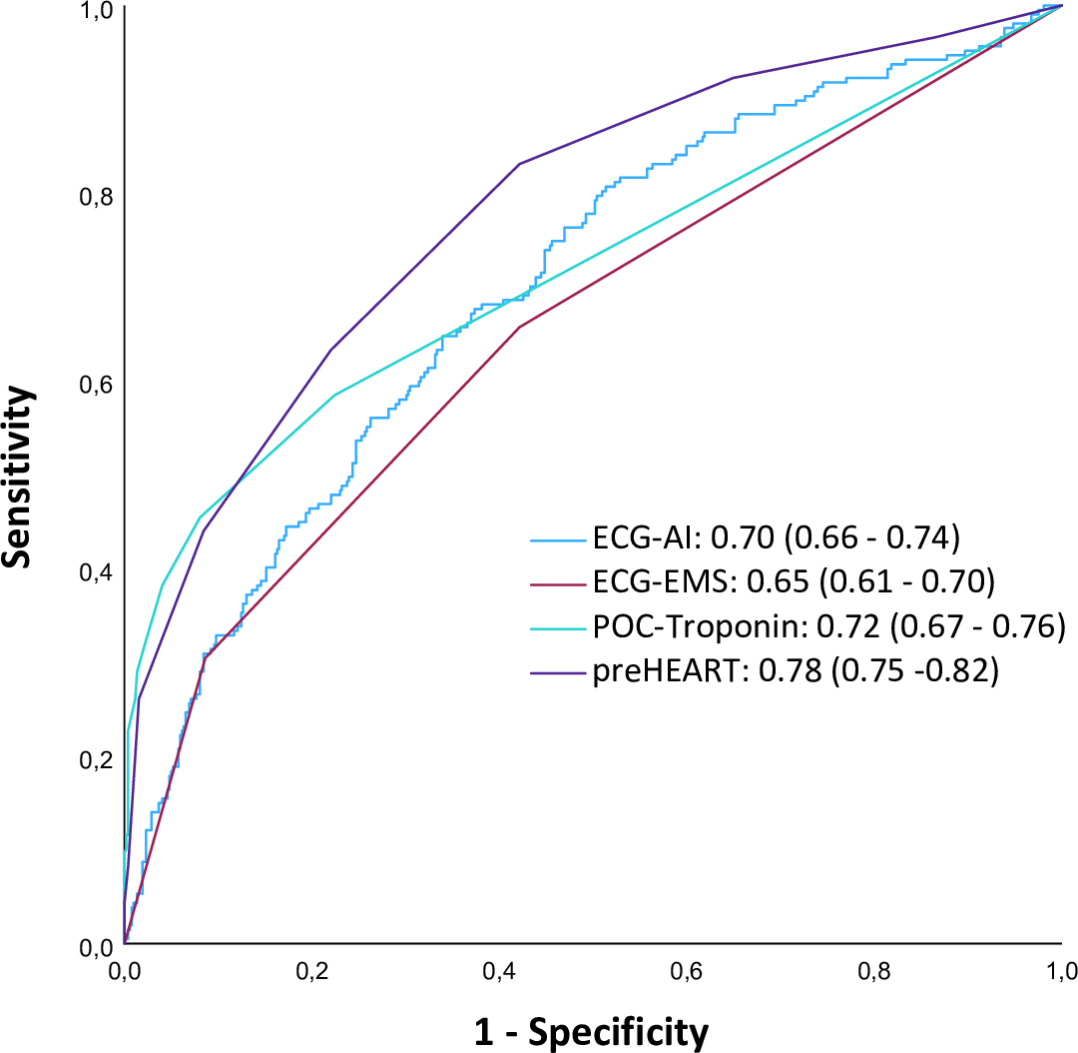
Receiver operating characteristic curves for prehospital diagnostic tools and corresponding area under the curve. ^*^ECG-AI versus ECG-EMS; p=0.045, ECG-AI versus POC-troponin; p=0.49, ECG-AI versus preHEART; p=0.001. CNN, convolutional neural network; ECG-AI, ECG interpretation by CNN; ECG-EMS, ECG interpretation by EMS paramedic; EMS, emergency medical services; POC, point-of-care; preHEART, prehospital History, ECG, Age, Risk factors and POC-troponin.

**Figure 3:**
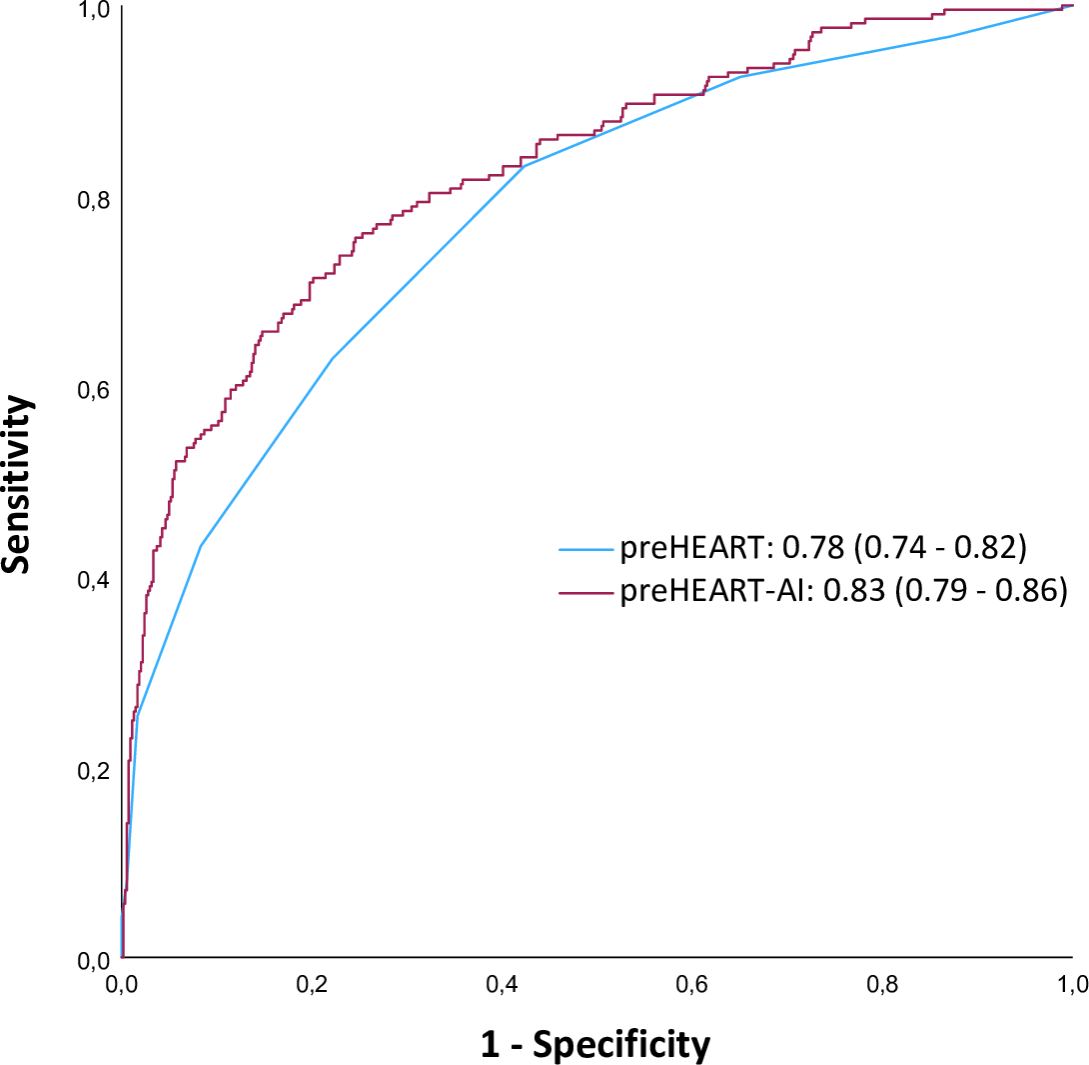
Receiver operating characteristic curves for preHEART and preHEART-AI and corresponding area under the curve. ^*^preHEART versus preHEART-AI; p<0.001. CNN, convolutional neural network; ECG-AI, ECG interpretation by CNN; ECG-EMS, ECG interpretation by EMS paramedic; EMS, emergency medical services; preHEART, prehospital History, ECG-EMS, Age, Risk factors and POC-troponin; preHEART-AI, prehospital History, ECG-AI, Age, Risk factors and POC-troponin.

Compared with ECG-EMS, ECG-AI had a better overall diagnostic performance (AUROC 0.70 (0.66 to 0.74) vs AUROC 0.65 (0.61 to 0.70), p=0.045) for diagnosing NSTE-ACS. Comparing ECG-AI and POC-troponin I showed no difference in overall diagnostic performance (AUROC 0.70 (0.66 to 0.74) vs AUROC 0.72 (0.67 to 0.76), p=0.49). The overall diagnostic performance of the preHEART score was 0.78 (0.74 to 0.82) which was superior compared with ECG-AI (p=0.001) (figure 2). After implementing ECG-AI into the preHEART score, the overall diagnostic performance of the preHEART-AI was improved (AUROC 0.83 (0.79 to 0.86), p<0.001) compared with the original preHEART score (figure 3).

To gain more insight into the differences between ECG-EMS and ECG-AI, we transformed the continuous ECG-AI parameter into three risk categories (low-intermediate-high), mirroring the risk categories used in ECG-EMS as part of the preHEART score. ECG-AI demonstrated statistically significantly higher NPV and sensitivity compared with ECG-EMS (p<0.001) in the lowest risk category. No differences were found between ECG-AI and ECG-EMS regarding specificity and PPV of the highest risk category (p=0.27) (table 2, online supplemental tables S4 and S5).

**Table 2:** Diagnostic performance of ECG-EMS and ECG-AI

	ECG-EMS	ECG-AI
Sensitivity* (%)	65.7 (58.9 to 72.1)	80.3 (74.3 to 85.4)
Specificity† (%)	91.3 (88.6 to 93.6)	89.8 (87.0 to 92.3)
NPV* (%)	81.2 (78.0 to 84.1)	86.6 (83.0 to 89.6)
PPV† (%)	57.7 (49.3 to 65.8)	56.4 (48.6 to 63.9)

* For the low-risk group.

† For the high-risk group.

CNN, convolutional neural network; ECG-AI, ECG interpretation by CNN; ECG-EMS, ECG interpretation by EMS paramedic; EMS, emergency medical services; NPV, negative predictive value; PPV, positive predictive value.

## Discussion

**Figure 4:**
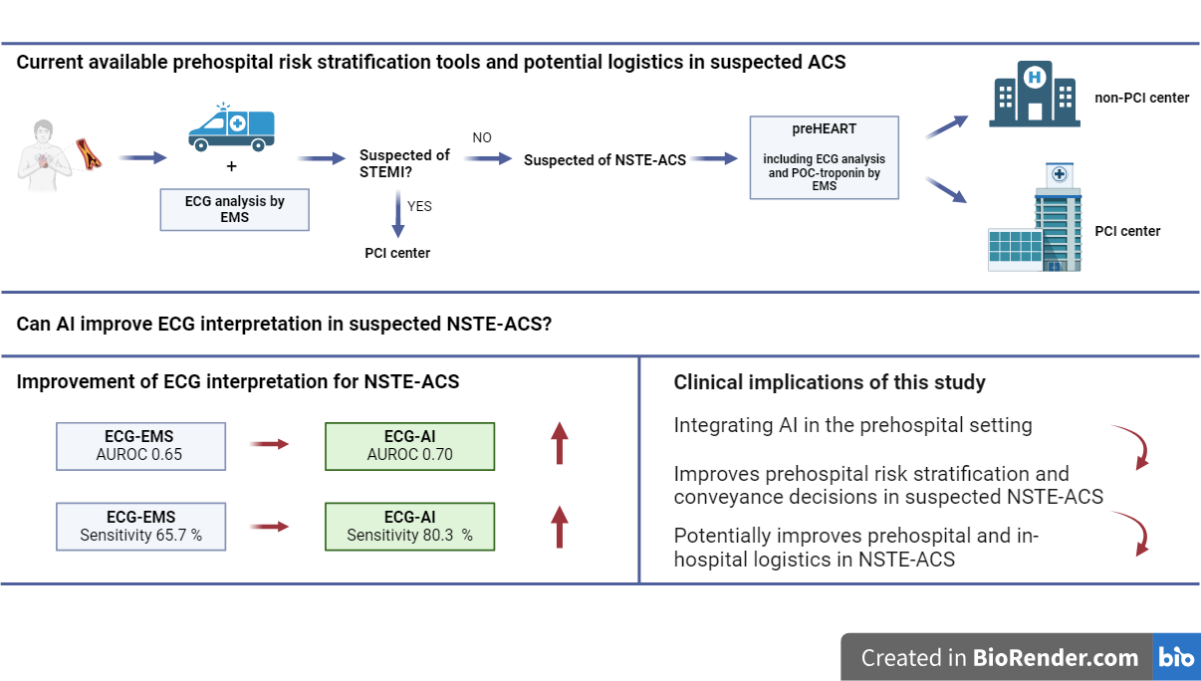
Central image. ACS, acute coronary syndrome; AI, artificial intelligence; AUROC, area under the curve; ECG-AI, ECG interpretation by CNN; ECG-EMS, ECG interpretation by EMS paramedic; EMS, emergency medical services; NSTE-ACS, non-ST-elevation ACS; preHEART, prehospital History, ECG, Age, Risk factors and POC-troponin; STEMI, ST-segment elevation myocardial infarction.

In this study, we developed and validated a CNN-based AI model for diagnosing NSTE-ACS in chest pain patients in the prehospital setting, using only the ECG as input. The AI model outperformed ECG interpretation by EMS paramedics, especially in identifying patients at low risk for having NSTE-ACS. Compared with a prehospital clinical risk score, the AI model did not match the diagnostic performance of the preHEART score. However, clinical risk scores could benefit from incorporating AI-enhanced ECG interpretation (figure 4).

To the best of our knowledge, this study stands out from prior research endeavours by our development and validation of a CNN-based AI model to classify NSTE-ACS based on the ECG. Moreover, we have undertaken a comparative analysis to assess the diagnostic performance of our model to the presently available prehospital diagnostic tools. Using AI-assisted interpretation of the ECG, the EMS paramedic is better able to differentiate between NSTE-ACS and non-cardiac chest pain compared with relying on their own interpretation. Especially in identifying patients at low risk for having NSTE-ACS, the AI model outperformed the EMS paramedic. Correctly identifying patients who are at low risk for having NSTE-ACS is crucial for optimal triage in the prehospital setting. Recent studies have shown that these low-risk patients could potentially be left at home or transferred to a general practitioner, leading to less ED overcrowding and lower healthcare costs.^11^ Nevertheless, the PPV of the AI model is only modest and does not outperform ECG interpretation by EMS paramedics. This is likely due to the heterogeneity within the NSTE-ACS population, as some patients present with significant ischaemic changes on the ECG, while others show minimal or no changes. This variability makes consistent risk stratification based on the ECG findings challenging. In addition to improving diagnostic performance, using a model for ECG interpretation in the prehospital setting addresses the issue of inter-operator variability. Inter-operator variability is common among medical personnel when scoring the ECG and is highly dependent on experience and training.^6 7 31^ EMS paramedics receive training aimed at recognising a STEMI, but no training in further assessing more subtle ECG changes such as minimal ST-depression and T-wave inversion.^32 33^ However, these changes are important to recognise and score in a clinical risk score.^5 15^

Other studies, which used a machine learning-based AI model for prehospital ECG diagnosis in patients with chest pain, demonstrated better overall diagnostic performance compared with our AI model.^22 23^ However, these studies were aimed at a study population with a high prevalence of occlusive myocardial infarction (OMI), which could explain the differing levels of diagnostic performance.^22^ In patients with OMI, ischaemia is persistent, making ECG abnormalities more pronounced and therefore more easily recognised by medical experts and AI models alike.^22 34^ Moreover, in our study population, we included a more selected population as patients with suspected NSTE-ACS with very high risk criteria according to the ESC and AHA/ACC guidelines (eg, haemodynamic unstable, cardiogenic shock, ongoing chest pain refractory to medical treatment) in the EMS setting were excluded.^1 2^ Therefore, by excluding very high-risk patients on one hand and including a substantial number of ACS patients with non-occluded myocardial infarction on the other, we likely encounter more subtle, if any, ECG abnormalities, which also limit detection by an AI model.

Previous studies have shown that clinical risk scores have the best diagnostic performance for prehospital risk stratification in suspected NSTE-ACS patients, and our results confirm these findings.^5 8^ Our study shows that using single prehospital diagnostic tools, such as ECG and POC-troponin, both by human interpretation and AI, is not sufficient when used alone in the prehospital setting to identify NSTE-ACS patients.^35^ However, combining clinical parameters in a clinical risk score significantly improves the overall diagnostic performance. Additionally, incorporating AI into clinical risk scores can enhance diagnostic performance even further.

### Limitations

This study suffers from limitations inherent to the study design. First, we used only DeepArrNet to develop the CNN-based model. We did not investigate alternative architectures to determine whether they might yield a model with superior diagnostic performance. Second, the AI model was developed within a study population of modest size. Nonetheless, despite this limited cohort, we observed a noteworthy enhancement in ECG interpretation accuracy in patients at low risk for having NSTE-ACS. It is anticipated that further refinement can be achieved through the training of the model in larger populations, thereby augmenting its overall performance. A third limitation is that the ECG-AI score is only validated in a very specific cohort of patients within the EMS setting. The model’s reliability in patients without chest pain or in other settings remains unknown. Although the AI model demonstrated superior diagnostic performance compared with ECG interpretation by EMS paramedics, its effectiveness should be evaluated in other settings and healthcare systems with potentially different chest pain populations to determine whether this performance advantage persists.

## Conclusion

Integrating AI in prehospital ECG interpretation improves the identification of patients at low risk for having NSTE-ACS. Nonetheless, clinical risk scores currently yield the best diagnostic performance and their accuracy could be further enhanced through artificial intelligence. Our results pave the way for new studies focused on exploring the role of AI in prehospital risk-stratification efforts.

## Supplementary material

10.1136/bmjhci-2024-101292 online supplemental file 1

## Data Availability

Data are available upon reasonable request.

## References

[1] Byrne RA, Rossello X, Coughlan JJ (2023). 2023 ESC Guidelines for the management of acute coronary syndromes. Eur Heart J.

[2] Writing Committee M (2021). 2021 AHA/ACC/ASE/CHEST/SAEM/SCCT/SCMR Guideline for the Evaluation and Diagnosis of Chest Pain: A Report of the American College of Cardiology/American Heart Association Joint Committee on Clinical Practice Guidelines. J Am Coll Cardiol.

[3] Stengaard C, Sørensen JT, Rasmussen MB (2016). Prehospital diagnosis of patients with acute myocardial infarction. Diagnosis (Berl).

[4] Dawson LP, Smith K, Cullen L (2022). Care Models for Acute Chest Pain That Improve Outcomes and Efficiency: JACC State-of-the-Art Review. J Am Coll Cardiol.

[5] Sagel D, Vlaar PJ, van Roosmalen R (2021). Prehospital risk stratification in patients with chest pain. Emerg Med J.

[6] Niven WGP, Wilson D, Goodacre S (2018). Do all HEART Scores beat the same: evaluating the interoperator reliability of the HEART Score. Emerg Med J.

[7] Snavely AC, Mahler SA, Hendley NW (2022). Prehospital Translation of Chest Pain Tools (RESCUE Study): Completion Rate and Inter-rater Reliability. West J Emerg Med.

[8] Demandt JPA, Zelis JM, Koks A (2022). Prehospital risk assessment in patients suspected of non-ST-segment elevation acute coronary syndrome: a systematic review and meta-analysis. BMJ Open.

[9] Stopyra JP, Snavely AC, Smith LM (2020). Prehospital use of a modified HEART Pathway and point-of-care troponin to predict cardiovascular events. PLoS ONE.

[10] Tolsma RT, Fokkert MJ, van Dongen DN (2022). Referral decisions based on a pre-hospital HEART score in suspected non-ST-elevation acute coronary syndrome: final results of the FamouS Triage study. Eur Heart J Acute Cardiovasc Care.

[11] Camaro C, Aarts GWA, Adang EMM (2023). Rule-out of non-ST-segment elevation acute coronary syndrome by a single, pre-hospital troponin measurement: a randomized trial. Eur Heart J.

[12] Demandt J, Koks A, Sagel D (2024). Prehospital risk assessment and direct transfer to a percutaneous coronary intervention centre in suspected acute coronary syndrome. Heart.

[13] van Dongen DN, Tolsma RT, Fokkert MJ (2020). Pre-hospital risk assessment in suspected non-ST-elevation acute coronary syndrome: A prospective observational study. Eur Heart J Acute Cardiovasc Care.

[14] Fox KAA, Dabbous OH, Goldberg RJ (2006). Prediction of risk of death and myocardial infarction in the six months after presentation with acute coronary syndrome: prospective multinational observational study (GRACE). BMJ.

[15] Antman EM, Cohen M, Bernink PJ (2000). The TIMI risk score for unstable angina/non-ST elevation MI: A method for prognostication and therapeutic decision making. JAMA.

[16] van Steenbergen GJ, Demandt JPA, Schulz DN (2023). Direct admission versus interhospital transfer for revascularisation in non-ST-segment elevation myocardial infarction. Clin Cardiol.

[17] Attia ZI, Harmon DM, Behr ER (2021). Application of artificial intelligence to the electrocardiogram. Eur Heart J.

[18] Siontis KC, Noseworthy PA, Attia ZI (2021). Artificial intelligence-enhanced electrocardiography in cardiovascular disease management. Nat Rev Cardiol.

[19] Attia ZI, Friedman PA, Noseworthy PA (2019). Age and Sex Estimation Using Artificial Intelligence From Standard 12-Lead ECGs. Circ Arrhythm Electrophysiol.

[20] van de Leur RR, van Sleuwen MTGM, Zwetsloot P-PM (2024). Automatic triage of twelve-lead electrocardiograms using deep convolutional neural networks: a first implementation study. Eur Heart J Digit Health.

[21] Noseworthy PA, Attia ZI, Behnken EM (2022). Artificial intelligence-guided screening for atrial fibrillation using electrocardiogram during sinus rhythm: a prospective non-randomised interventional trial. Lancet.

[22] Al-Zaiti SS, Martin-Gill C, Zègre-Hemsey JK (2023). Machine learning for ECG diagnosis and risk stratification of occlusion myocardial infarction. Nat Med.

[23] Al-Zaiti S, Besomi L, Bouzid Z (2020). Machine learning-based prediction of acute coronary syndrome using only the pre-hospital 12-lead electrocardiogram. Nat Commun.

[24] Demandt JPA, Koks A, Haest R (2022). Prehospital triage of patients with suspected non-ST-segment elevation acute coronary syndrome: Rationale and design of the TRIAGE-ACS study. Contemp Clin Trials.

[25] Collet J-P, Thiele H (2020). The “Ten Commandments” for the 2020 ESC Guidelines for the management of acute coronary syndromes in patients presenting without persistent ST-segment elevation. Eur Heart J.

[26] Thygesen K, Alpert JS, Jaffe AS (2019). Fourth universal definition of myocardial infarction (2018). Eur Heart J.

[27] Mahmud T, Fattah SA, Saquib M (2020). DeepArrNet: An Efficient Deep CNN Architecture for Automatic Arrhythmia Detection and Classification From Denoised ECG Beats. IEEE Access.

[28] Apple FS, Murakami MM, Christenson RH (2004). Analytical performance of the i-STAT cardiac troponin I assay. Clin Chim Acta.

[29] Backus BE, Six AJ, Kelder JC (2010). Chest pain in the emergency room: a multicenter validation of the HEART Score. Crit Pathw Cardiol.

[30] DeLong ER, DeLong DM, Clarke-Pearson DL (1988). Comparing the areas under two or more correlated receiver operating characteristic curves: a nonparametric approach. Biometrics.

[31] Wu WK, Yiadom MYAB, Collins SP (2017). Documentation of HEART score discordance between emergency physician and cardiologist evaluations of ED patients with chest pain. Am J Emerg Med.

[32] Huitema AA, Zhu T, Alemayehu M (2014). Diagnostic accuracy of ST-segment elevation myocardial infarction by various healthcare providers. Int J Cardiol.

[33] Zègre-Hemsey JK, Patel MD, Fernandez AR (2020). A Statewide Assessment of Prehospital Electrocardiography Approaches of Acquisition and Interpretation for ST-Elevation Myocardial Infarction Based on Emergency Medical Services Characteristics. Prehosp Emerg Care.

[34] Aslanger EK, Yıldırımtürk Ö, Şimşek B (2020). DIagnostic accuracy oF electrocardiogram for acute coronary OCClUsion resuLTing in myocardial infarction (DIFOCCULT Study). Int J Cardiol Heart Vasc.

[35] Fischer JE, Bachmann LM, Jaeschke R (2003). A readers’ guide to the interpretation of diagnostic test properties: clinical example of sepsis. Intensive Care Med.

