# Supplementary material for: Towards prehospital risk stratification using deep learning for ECG interpretation in suspected acute coronary syndrome
Source: BMJ Health Care Inform. 2025 Jun 6;32(1):e101292. doi: 10.1136/bmjhci-2024-101292 (PMC12161418; doi:10.1136/bmjhci-2024-101292)
Supplement: online supplemental file 1 [file bmjhci-32-1-s001.pdf]

Supplemental material

Supplement to: Demandt JPA, Mast TP, van Beek KAJ D et al. Towards Prehospital Risk Stratification Using Deep Learning for ECG interpretation in Suspected Acute Coronary Syndrome

Table S1. Inclusion and exclusion criteria external validation cohort

Table S2. preHEART score

Table S3. Baseline characteristics internal training cohort

Table S4. Risk stratification according to ECG analysis by EMS

Table S5. Risk stratification according to ECG analysis by AI

Figure S1. Development and Validation of a Deep Learning Model for Diagnosing NSTE-ACS

Figure S2. Python's Keras framework

Table S1. Inclusion and exclusion criteria external validation cohort

**Inclusion**
Chest pain suspected for NSTE-ACS
Age ≥ 18 years
Intention to transfer patient to Emergency Department
**Exclusion**
ST-segment elevation Acute Coronary Syndrome
Post resuscitation patients
Hemodynamic instability defined as Killip Class IV
Suspected other life treating pathology
Pregnancy
Abbreviations: NSTE-ACS; non-ST-segment elevation Acute Coronary Syndrome

Table S2. preHEART score

History	Highly suspicious	2
	Moderately suspicious	1
	Slightly suspicious	0
ECG	Significant ST-depression and/or negative T-waves	2
	Non-specific repolarization disturbances	1
	Normal	0
Age	≥ 70 years	2
	40 – 69 years	1
	≤ 39 years	0
Risk factor	Male	2
	Female	0
POC - Troponin I	0.05 – 50 ng/ml	2
	0.03 – 0.04 ng/ml	1
	0.00 – 0.02 ng/ml	0
Total score:<br>0-3: low risk for having NSTE-ACS<br>4-7: intermediate risk for having NSTE-ACS<br>8-10: high risk for having NSTE-ACS		
Abbreviations: POC; Point-of-Care		

Table S3. Baseline characteristics internal training cohort

	Internal training cohort (n= 4891)
Age (years), mean ± SD	63 ± 15
Female sex, n (%)	2152/4891 (44)
NSTE-ACS, n (%)	1239/4891 (25)
Abbreviations: NSTE-ACS; non-ST-elevation ACS	

Table S4. Risk stratification according to ECG analysis by EMS

	**NSTE-ACS +**	**NSTE-ACS -**	**Total**
**Low risk**	73	312	385
**Intermediate risk**	75	182	257
**High risk**	65	47	112
**Total**	213	541	754
Abbreviations: ECG; electrocardiogram, EMS; Emergency Medical Services, NSTE-ACS; Non-ST-Elevation Acute Coronary Syndrome, low risk; normal ECG, intermediate risk; abnormal ECG with repolarization abnormalities, high risk; ECG with specific ischemic abnormalities			

Table S5. Risk stratification according to ECG analysis by AI

	**NSTE-ACS +**	**NSTE-ACS -**	**Total**
**Low risk**	42	269	311
**Intermediate risk**	99	217	316
**High risk**	72	55	127
**Total**	213	541	754
Abbreviations: ECG; electrocardiogram, AI; Artificial Intelligence, NSTE-ACS; Non-ST-Elevation Acute Coronary Syndrome, low risk; ECG-AI score < 0.04, intermediate risk; ECG-AI score 0.04 – 0.29, high risk; ECG-AI score > 0.29			

Figure S1. Development and Validation of a Deep Learning Model for Diagnosing NSTE-ACS

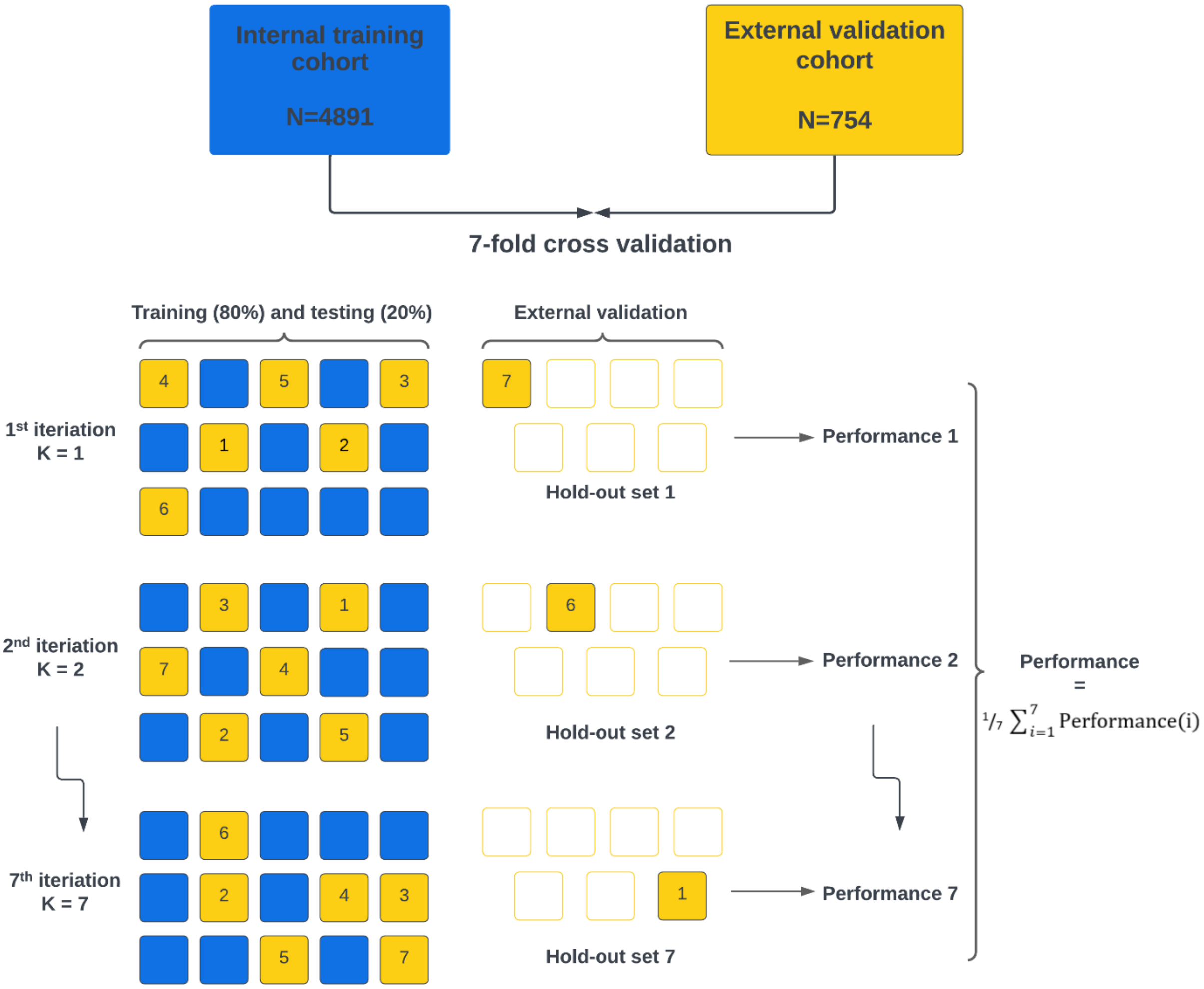

Step 1. Training and Testing Allocation: The internal training cohort and 6/7ths of the external validation cohort were combined to create training and testing datasets, with 80% of the combined dataset allocated for training and 20% for testing in each iteration.

Step 2. Purpose of External Validation Hold-Out Set: The unused 1/7th of the external validation cohort in each iteration served exclusively as a hold-out set for independent assessment of model performance. This ensured that external validation data remained untouched during the training process.

Figure S2. Python's Keras framework

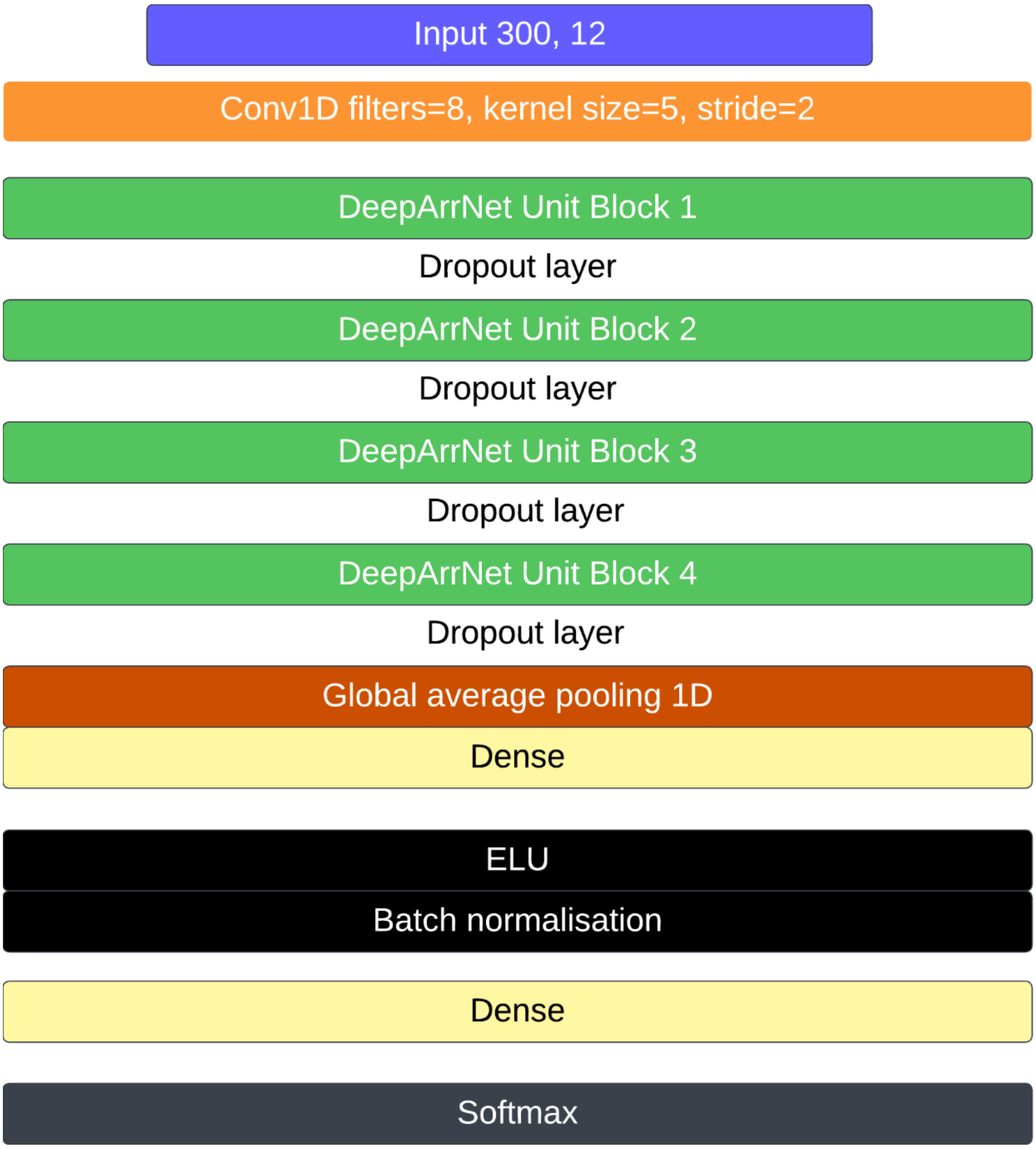

The median representation of the 10-second 12-lead standard ECG served as the model's input. Following a standard one-dimensional convolutional layer, the data was passed through several DeepArrnet unit blocks. Subsequently, a global average pooling layer and two dense layers were applied. The output was generated via a softmax layer, yielding a value between 0 and 1.
